# Supplementary figures and images for: Fibrotic extracellular matrix impacts cardiomyocyte phenotype and function in an iPSC-derived isogenic model of cardiac fibrosis
Source: Transl Res. 2024 Nov;273:58–77. doi: 10.1016/j.trsl.2024.07.003 (PMC11832458; doi:10.1016/j.trsl.2024.07.003)

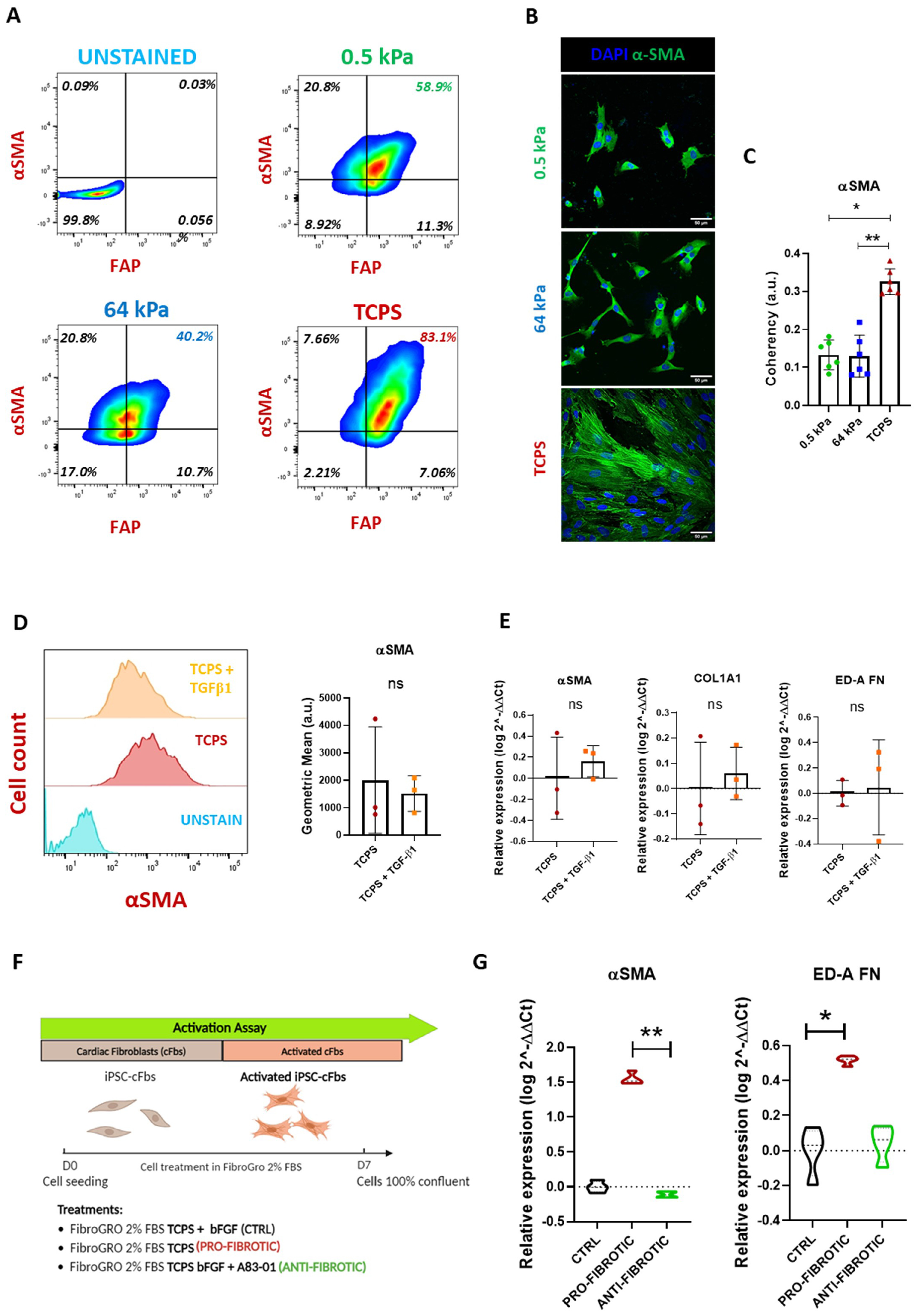

Supplement: Supplementary file 1 — Supplementary Fig. 1. Effect of substrate on iPSC-derived cardiac fibroblast activation. (A) Representative contour plots obtained by flow cytometry and describing the expression of Fibroblast Activated Protein (FAP) and alpha smooth muscle actin (α-SMA) in iPSC-derived cardiac fibroblasts cultured for 7 days on 0.5 kPa and 64 kPa substrates or on tissue culture polystyrene (TCPS). (B) Representative confocal images of iPSC-derived cardiac fibroblasts cultured for 7 days on 0.5 kPa and 64 kPa substrates or on tissue culture polystyrene (TCPS). The cells were stained for α-SMA (green) and the nuclei were counterstained with DAPI (blue) (scale bar: 50 µm). (C) Barplot representation of α-SMA fibres alignment coherency in iPSC-derived cardiac fibroblasts cultured for 7 days on 0.5 kPa and 64 kPa substrates or on tissue culture polystyrene (TCPS). The values are expressed as arbitrary units (a.u.). One-way ANOVA followed by Kruskal-Wallis test (N = 3; n = 6). *p < 0.05, ** p < 0.01, data presented as mean ± standard deviation. (D) Representative histogram plot (left) and its relative barplot representation (right) of α-SMA expression in iPSC-derived cardiac fibroblasts cultured for 7 days on tissue culture polystyrene (TCPS) alone or in the presence of TGFβ-1 as obtained by flow cytometry. The curves in the histogram plot indicate cell counts, while α-SMA values in the barplot are expressed as intensity geometric mean. One-way ANOVA followed by Kruskal-Wallis test (N = 3; n = 3). Data presented as mean ± standard deviation. (E) Barplot representation of the expression of the indicated genes in iPSC-derived cardiac fibroblasts cultured for 7 days on tissue culture polystyrene (TCPS) alone or in the presence of TGFβ-1 as obtained by RT-qPCR. The results are expressed as log 2^-(ΔΔCt) normalized to TCPS. Data normalized on GAPDH expression. One-way ANOVA followed by Kruskal-Wallis test (N = 3, n = 3). N.S.= non-significant. Data presented as mean ± standard deviation. (F) [file mmc1.jpg]

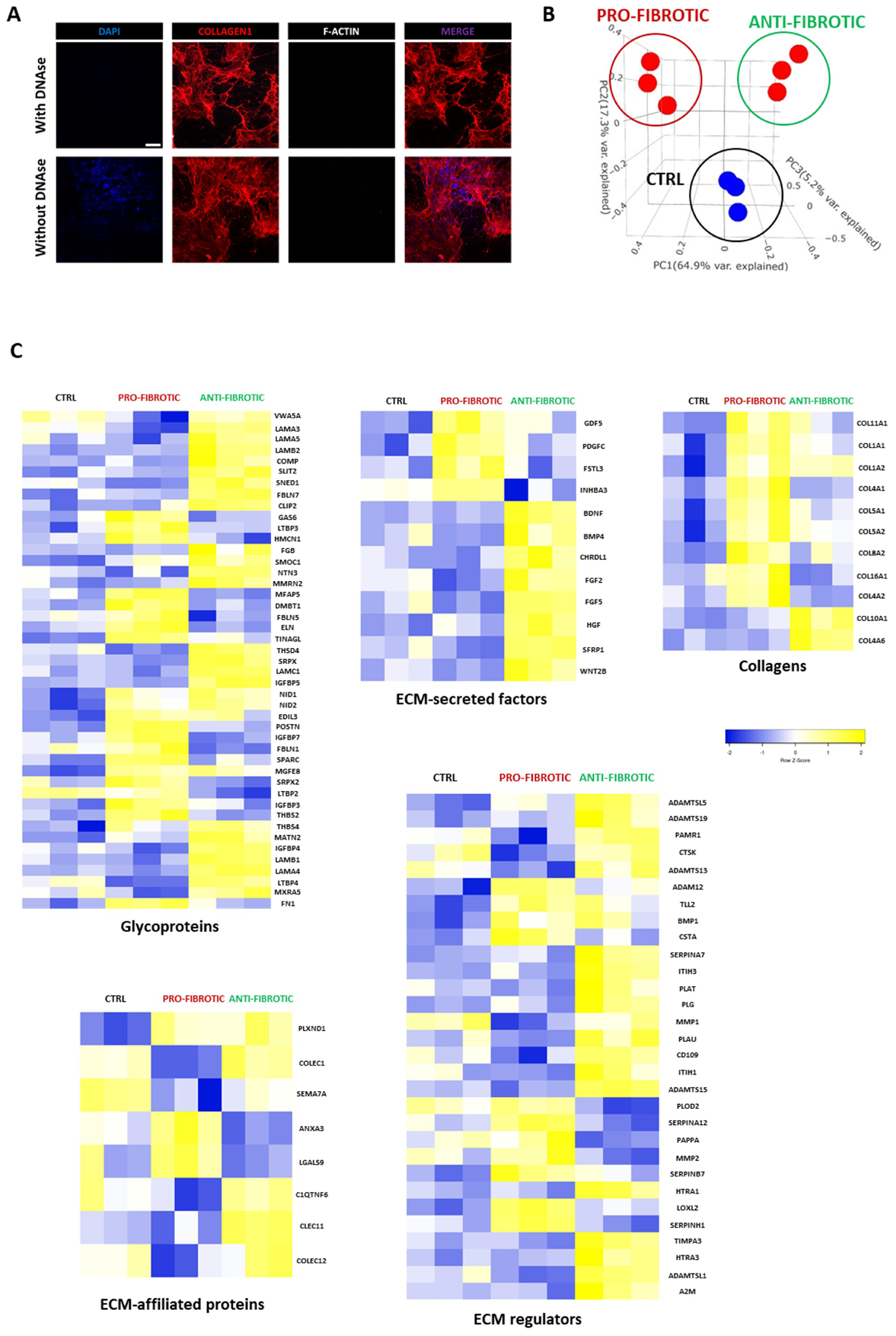

Supplement: Supplementary file 2 — Supplementary Fig. 2. Outcome of decellularization procedure and mass spectrometry characterization of the dECMs. (A) Representative confocal images of dECMs obtained in the presence (up) or absence (down) of DNAse treatment. Collagen I stained in red, F-actin in gray (absent), and nuclei counterstained with DAPI. Scale bar: 100µm. (B) 3D principal component analysis (PCA) of the protein composition of dECMs deposited by ctrl, pro- and anti-fibrotic iPSC-derived cardiac fibroblasts, as obtained by Mass Spectrometry. The analysis was performed via BIOJUPIES (124). (C) Heatmap representation of the indicated genes belonging to the annotated categories (glycoproteins, ECM regulators, ECM-affiliated proteins, ECM-secreted factors) found expressed in dECMs deposited by ctrl, anti-fibrotic and pro-fibrotic iPSC-derived cardiac fibroblasts. [file mmc2.jpg]

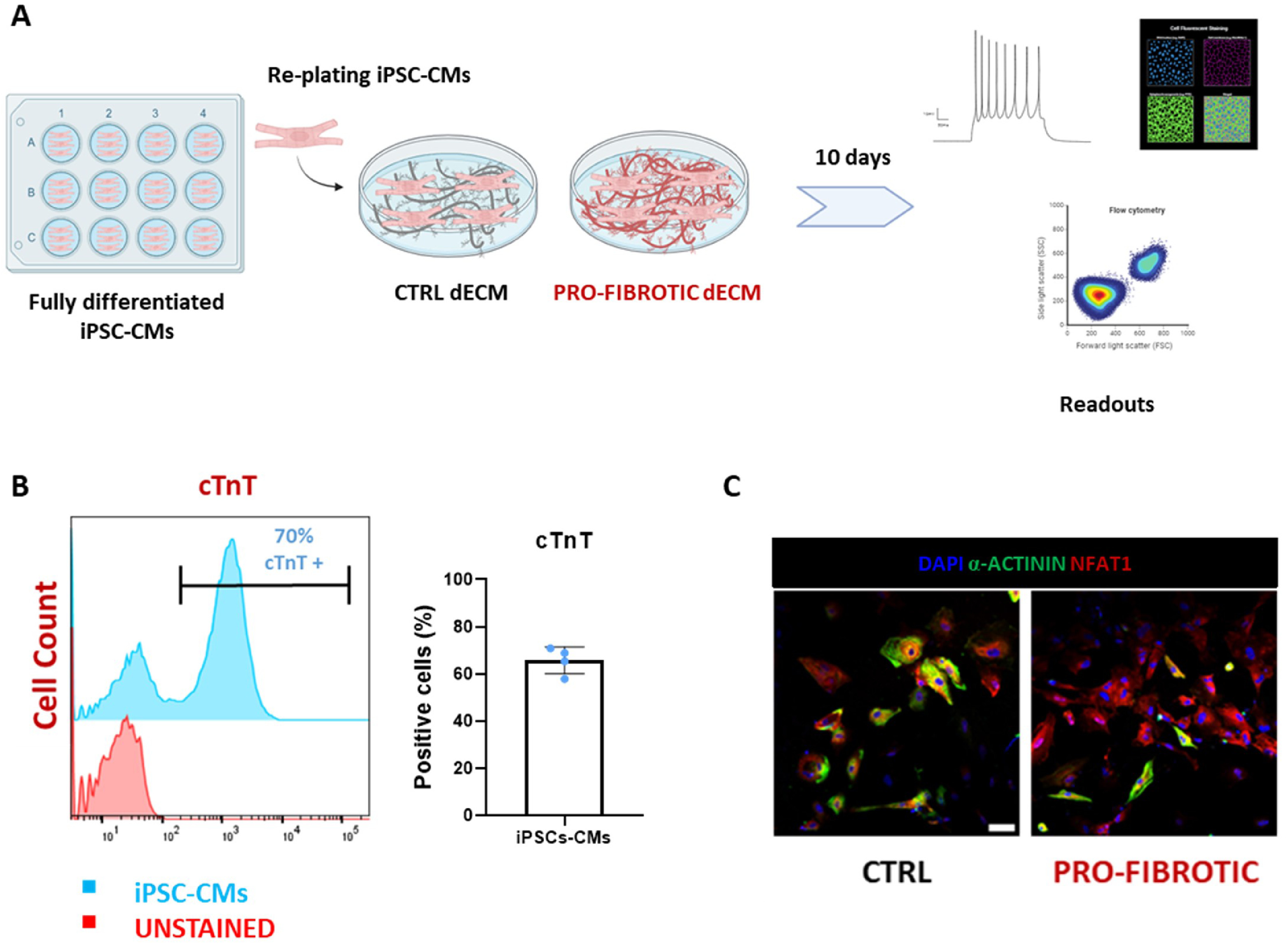

Supplement: Supplementary file 3 — Supplementary Fig. 3. Characterization of iPSC-derived cardiomyocytes cultured onto the dECMs deposited by iPSC-derived cardiac fibroblasts. (A) Schematic representation of the protocol adopted in the study. (B) Representative histogram plot (left) and relative barplot quantification (right) of the percentage of cells expressing cTnT in iPSC-derived cardiomyocytes at day 20 of differentiation. The data were obtained by flow cytometry for 4 independent experiments (N = 4, n = 4). Data presented as mean ± standard deviation. (C) Representative confocal images of iPSC-derived cardiomyocytes cultured on ctrl and pro-fibrotic dECM and stained for α-actinin (green) and NFAT1 (red). The nuclei were counterstained with DAPI (blue) (Scale bar: 50 µm). [file mmc3.jpg]

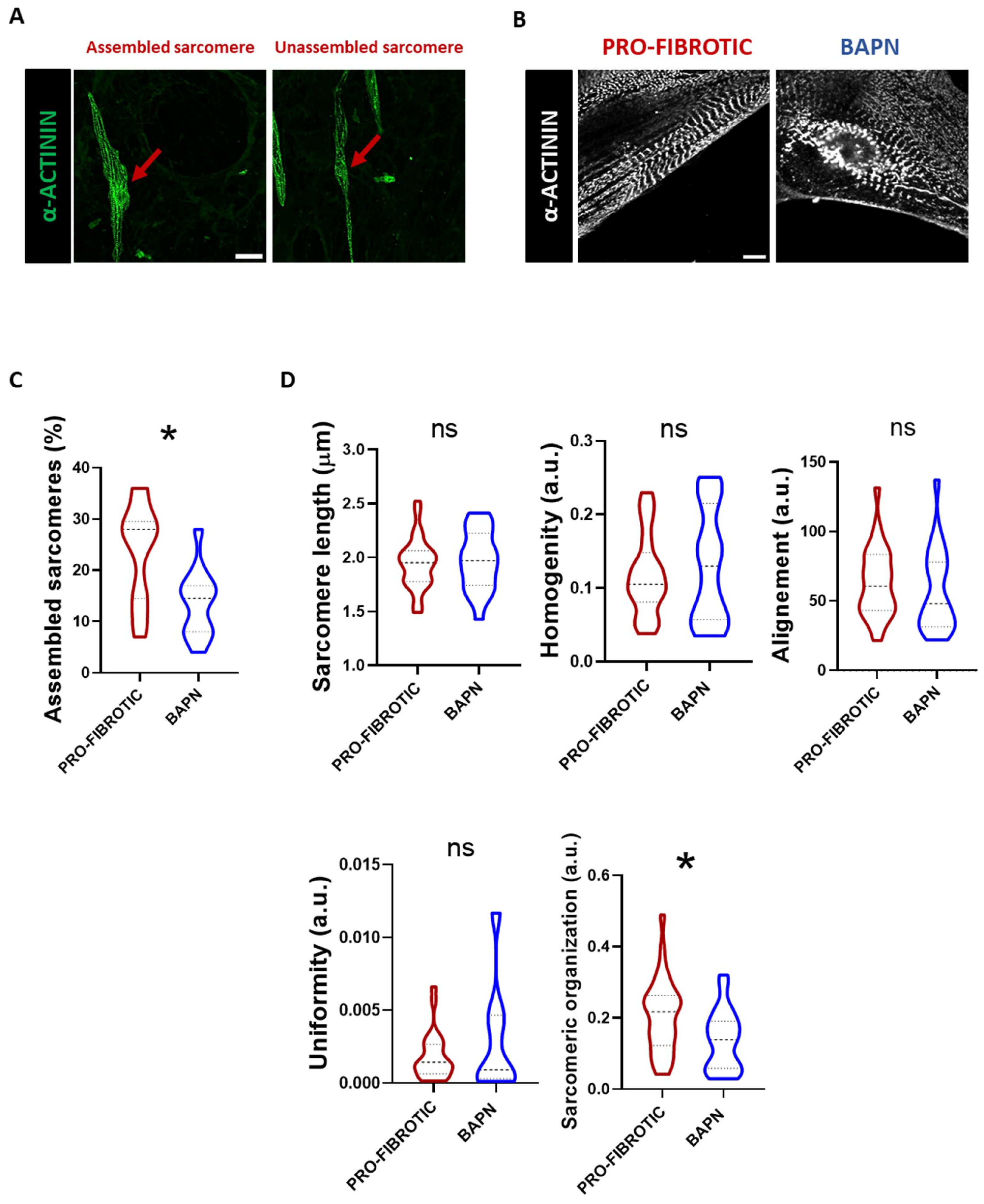

Supplement: Supplementary file 4 — Supplementary Fig. 4. Effects of dECM collagen crosslinking inhibition on cardiomyocytes sarcomere organisation. (A) Representative confocal pictures of cardiomyocytes stained for α-sarcomeric actinin (green) and used to discriminate between cardiomyocytes with assembled (left) or unassembled sarcomeres (right). Scale bar: 30µm. (B) High-resolution confocal image representing (white) in cardiomyocytes cultured on pro-fibrotic dECM treated (right) or not (left) with BAPN. Scale bar: 5µm. (C) Violin plot representation of the data obtained α-sarcomeric actinin from the analysis of cardiomyocytes characterized by assembled sarcomere following the interaction with either pro-fibrotic or BAPN-treated dECM. The data are presented as percentage ± standard deviation of α-sarcomeric actinin-positive cells scored by two independent blinded operators. Non-parametric Mann-Whitney t-test (N = 3; n = 12). *= p < 0.05. (D) Violin plots representation of the quantification of sarcomere length (N = 3; n = 29 for pro-fibrotic, n = 22 for BAPN), homogeneity (N = 3; n = 29 for pro-fibrotic, n = 22 for BAPN), alignment index (N = 3; n = 27 for pro-fibrotic, n = 22 for BAPN), uniformity (N = 3; n = 24 for pro-fibrotic, n = 18 for BAPN), and organization score (N = 3; n = 29 for pro-fibrotic, n = 22 for BAPN) in iPSC-derived cardiomyocytes cultured either on pro-fibrotic or BAPN-treated dECM. N.S.= non-significant; *= p < 0.05. Data presented as mean ± standard deviation. [file mmc4.jpg]

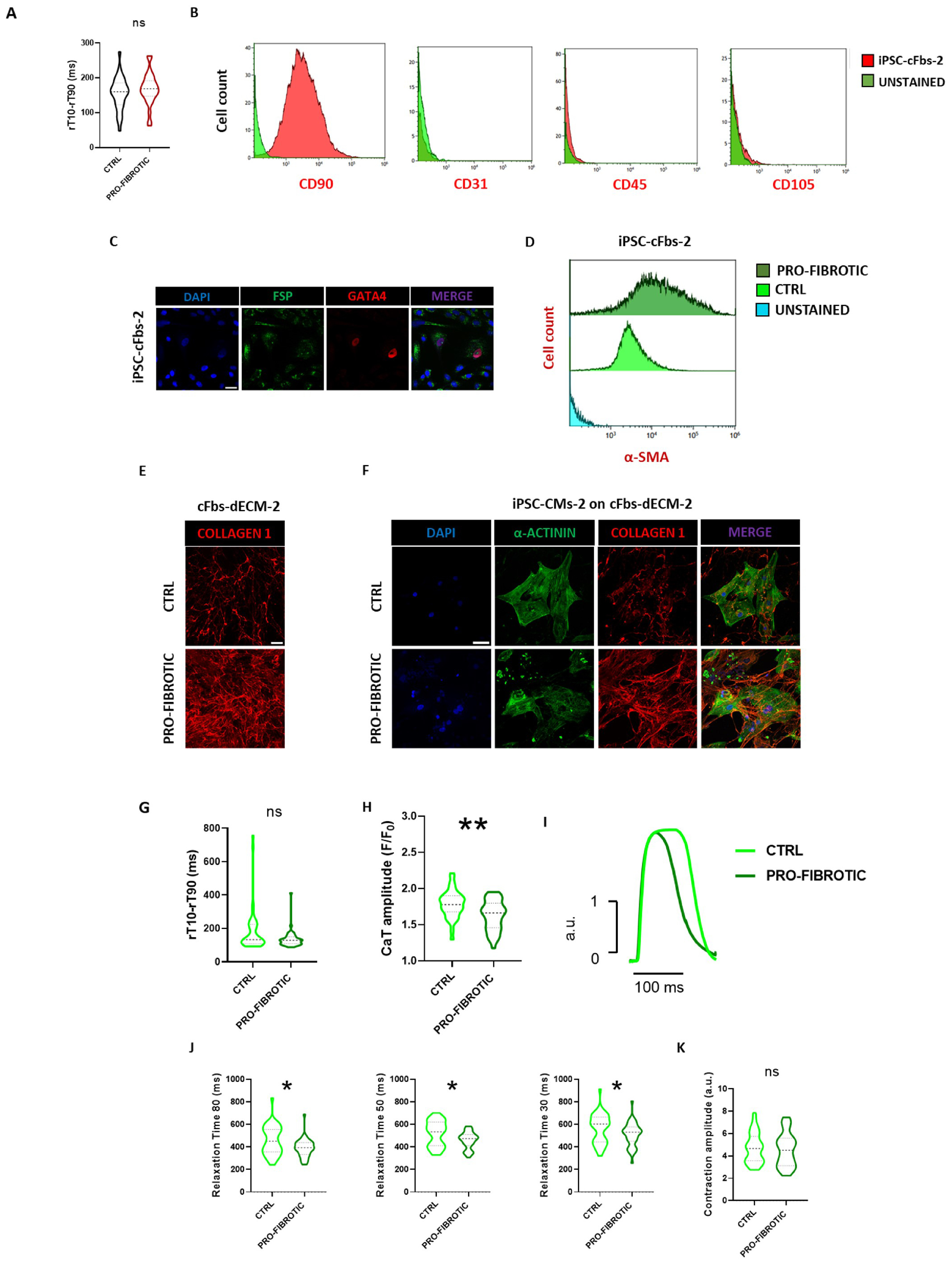

Supplement: Supplementary file 5 — Supplementary Fig. 5. Calcium uptake in iPSC-CMs and generation of a second isogenic model using the iPSC2 cell line. (A) Violin plot representation of the calcium uptake time (rT90-rT10) in iPSC-derived cardiomyocytes cultured onto dECMs deposited from iPSC-derived cardiac fibroblasts exposed or not to pro-fibrotic stimuli. Non-parametric Mann-Whitney test. (N = 3; n =30 for control, n = 28 for pro-fibrotic). N.S.= non-significant. Data are presented as mean ± standard deviation. (B) Representative flow cytometry histogram plots for fibroblast (CD90), endothelial (CD31), immune (CD45), and mesenchymal (CD73) cell markers. In red is indicated the stained iPSC-cFbs-2, while in green is the unstained control. (C) Representative confocal images representing iPSC-cFbs-2 stained for FSP (green), GATA4 (red). Nuclei counterstained with DAPI (blue). Scale bar: 30µm. (D) Representative flow cytometry histogram showing the expression of α-SMA in iPSC-cFbs-2 cultured either in pro-fibrotic (dark green) or in control (light green) condition. In light blue is marked the unstained control. (E) Representative confocal picture of dECM derived from control and pro-fibrotic iPSC-cFbs-2 stained for collagen-1 (red). Scale bar: 50µm. (F) Confocal pictures representing iPSC-CMs-2 stained for α-sarcomeric actinin (green) and cultured for long-term on ctrl and pro-fibrotic dECM, marked for collagen-1 (red). Nuclei counterstained in blue with DAPI. Scale bar: 30µm. (G) Violin plot representation of the calcium uptake time (rT90-rT10) in iPSC-CMs-2 cultured onto dECMs deposited from iPSC-cFbs-2 exposed or not to pro-fibrotic stimuli (N = 4; n =40). Non-parametric Mann-Whitney test. N.S.= non-significant. Data presented as mean ± standard deviation. (H) CaT amplitude measured in iPSC-CMs-2 cultured on ctrl and pro-fibrotic dECM-2 (N = 4; n = 37). Statistical analysis performed by non-parametric Mann-Whitney t-test. ** p < 0.01. Data presented as mean ± standard deviation. (I) Representative [file mmc5.jpg]
